# Gradenigo’s syndrome secondary to chronic otitis media on a background of previous radical mastoidectomy: a case report

**DOI:** 10.1186/1752-1947-8-217

**Published:** 2014-06-23

**Authors:** Yuvatiya Plodpai, Siriporn Hirunpat, Weerawat Kiddee

**Affiliations:** 1 Department of Otolaryngology, Faculty of Medicine, Prince of Songkla University, Hatyai, Songkhla 90110, Thailand; 2 Department of Radiology, Faculty of Medicine, Prince of Songkla University, Hatyai, Songkhla 90110, Thailand; 3 Department of Ophthalmology, Faculty of Medicine, Prince of Songkla University, Hatyai, Songkhla 90110, Thailand

**Keywords:** Gradenigo’s syndrome, Mastoidectomy, Petrous apicitis

## Abstract

**Introduction:**

Gradenigo’s syndrome is nowadays a rare condition characterized by a triad of otorrhea, facial pain with trigeminal nerve involvement and abducens nerve palsy. Most cases are caused by medial extension of acute otitis media into a pneumatized petrous apex and surgical drainage is usually the treatment of choice. We present a case highlighting the pathological mechanism of this disease, demonstrate rare radiological findings associated with this patient, and showcase successful medical treatment without surgical intervention.

**Case presentation:**

A 63-year-old Thai man presented with complete Gradenigo triad as a complication of chronic otomastoiditis in spite of clinical history of previous radical mastoidectomy and a nonpneumatization of the petrous apex. Magnetic resonance imaging showed abnormal prominent enhancement at the roof of his right temporal bone, and the dura overlying the floor of right middle cranial fossa and right cavernous sinus. Magnetic resonance imaging also detected right petrous apicitis. With the use of intravenous antibiotics and topical antibiotic eardrops, recovery was observed within 5 days with complete resolution within 2 months.

**Conclusions:**

Although there is little evidence to support the use of medical therapy in the treatment of Gradenigo’s syndrome resulting from chronic ear disease, we here demonstrate successful conservative treatment of Gradenigo’s syndrome following chronic otitis media in a patient who underwent previous radical mastoidectomy.

## Introduction

Gradenigo’s syndrome (GS) was first described in 1904 by Guiseppe Gradenigo [1]. It is defined as a clinical triad of otitis media, severe pain originating from the trigeminal nerve, and ipsilateral sixth cranial nerve palsy. The syndrome is an exceedingly rare complication of chronic otitis media in the era of the widespread use of antibiotics and easily accessible health-care services. Classically, the symptoms are related to inflammation of the petrous apex of the temporal bone, a condition termed petrous apicitis, which is usually caused by medial extension of acute otitis media into a pneumatized petrous apex, located near the trigeminal ganglion and sixth cranial nerve. To the best of our knowledge, GS developing in a patient with a previous radical mastoidectomy and nonpneumatization of the petrous apex has never been reported. We highlight a different pathological disease mechanism and demonstrate rare radiologic findings. A dramatic response was achieved by administering intravenous antibiotics following nonsurgical management.

## Case presentation

**Figure 1:**
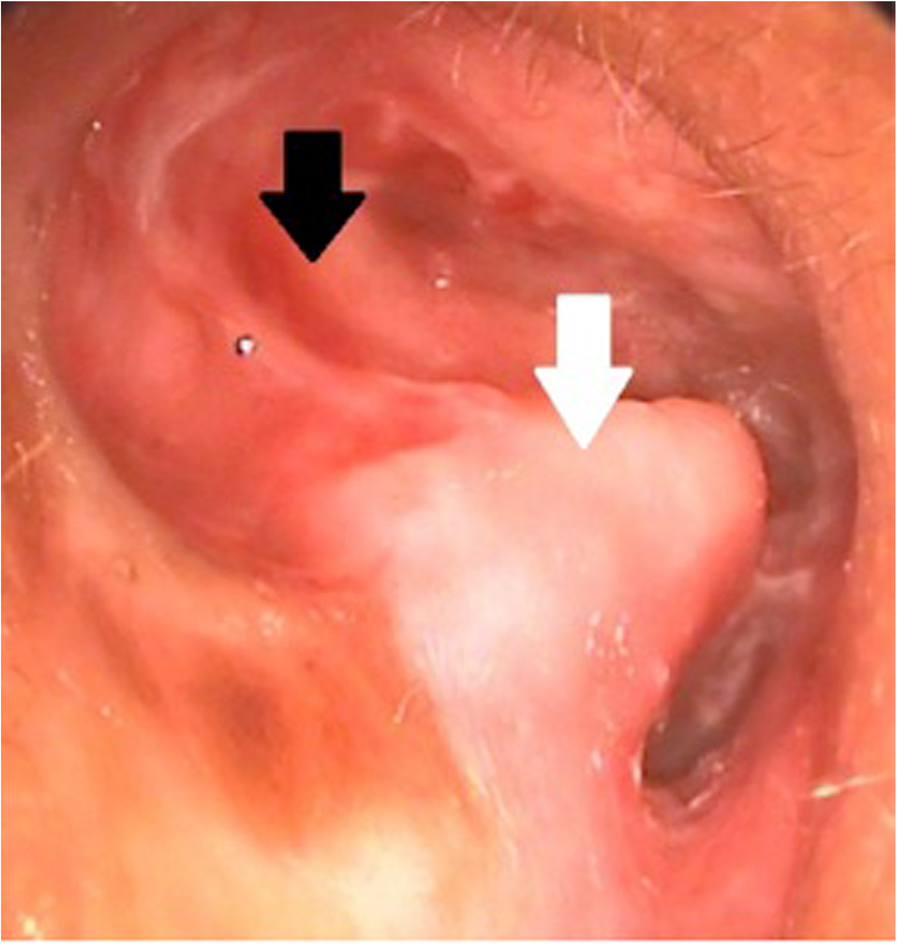
Otoscopic view showing an inflamed right mastoid cavity (black arrow) with a high facial ridge (white arrow) post-radical mastoidectomy.

A 63-year-old Thai man with a history of right-sided otorrhea presented with a 2-day history of right abducens nerve palsy and severe headache. His headache began 1 month ago concomitant with right-sided otorrhea. At that time, his otorrhea improved temporarily after taking empirical systemic and otic antibiotics, but the headache did not. Five days prior to presentation, he experienced worsening headache, nausea, and vomiting. Emergency computed tomography (CT) was initially reported as an unremarkable study. Three days later, he developed binocular diplopia in a primary gaze position that worsened when looking to a right gaze. The constant headache had begun with ipsilateral pain in his right frontal and retro-orbital area radiating to his whole head. His medical history was unremarkable with the exception of a right ear radical mastoidectomy performed 30 years previously. He was lost to follow-up and still had persistent right ear discharge.An otoscopy of his right ear revealed mucoid discharge and granulation tissue occupying the middle ear and mastoid cavity, which was inflamed with a high facial ridge (Figure 1, see white arrow). His left ear contained an intact tympanic membrane and a narrowing of the external ear canal. Ophthalmologic examination revealed an isolated right-sided abducens nerve palsy. All other neurological examination findings were unremarkable.

**Figure 2:**
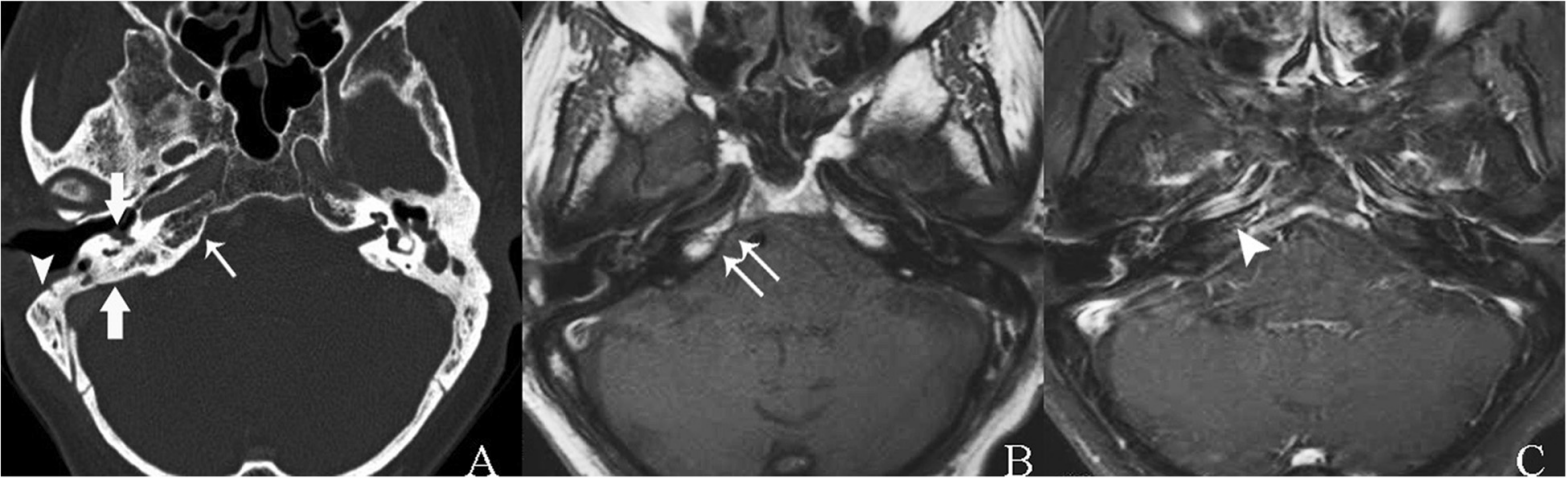
**Axial computed tomography and magnetic resonance imaging of temporal bone. A**. Thin-slice bone algorithm non-contrast computed tomography revealed partial opacified right middle ear cavity and residual mastoid air cells (thick arrows) as a result of chronic otomastoiditis, and post-mastoidectomy status seen as localized defect along the anterolateral wall of right mastoid air cells (arrowhead). Poor pneumatization of right petrous apex (arrow) was noted and was later confirmed by magnetic resonance imaging. **B**. Spin echo T1-weighted magnetic resonance imaging demonstrated high signal intensity of fatty marrow (arrows), as a normal variant. **C**. Postgadolinium-pentetic acid spin echo T1-weighted with fat suppression magnetic resonance imaging reveals subtle evidence of right petrous apicitis seen as slightly asymmetrical prominent enhancement as compared to the normal left side (arrowhead).

**Figure 3:**
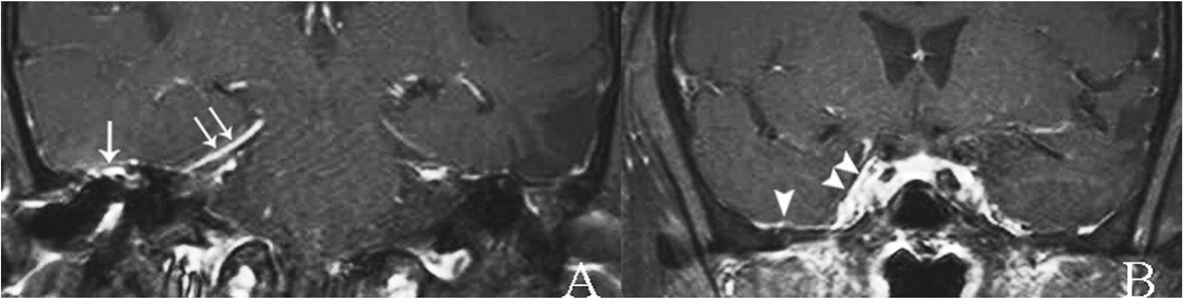
**Coronal postgadolinium-pentetic acid spin echo T1-weighted with fat suppression magnetic resonance imaging. A**. Abnormal prominent enhanced roof of the middle ear cavity (arrow) and thickened enhanced anteromedial part of right tentorial cerebelli (arrows). **B**. Abnormal prominent enhanced floor of right middle cranial fossa (arrowhead) and right cavernous sinus (arrowheads).

**Figure 4:**
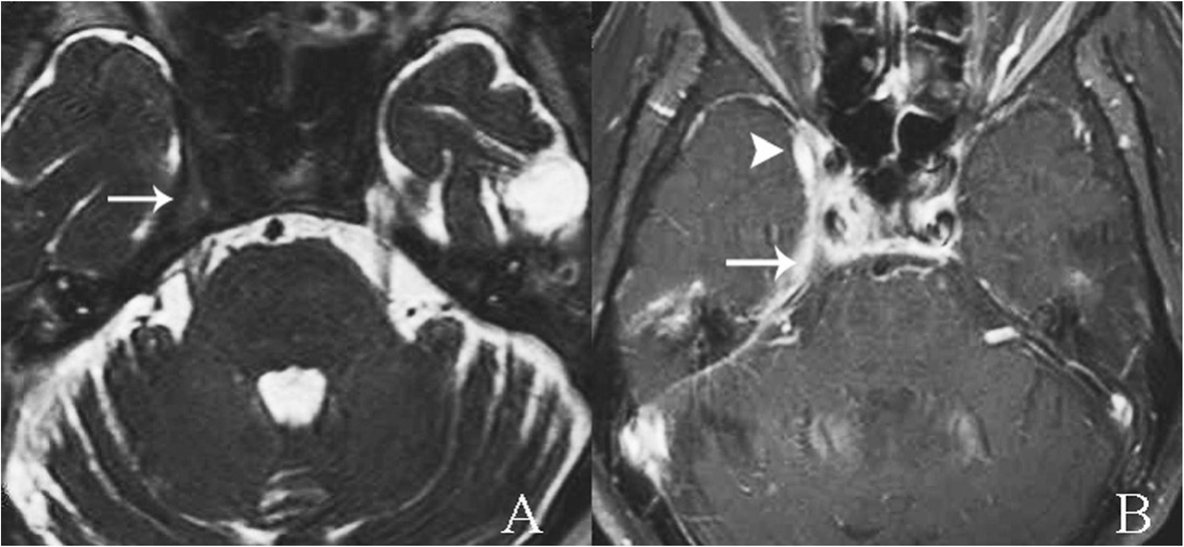
**Axial magnetic resonance imaging at the level of the cavernous sinuses. A**. Thin-slice axial T2-weighted images obtained by three-dimensional driven equilibrium sequence revealed edematous right trigeminal ganglion (arrow). **B**. Postgadolinium-pentetic acid spin echo T1-weighted with fat suppression magnetic resonance imaging of temporal bone. Edematous right trigeminal ganglion within right Meckel’s cave (arrow) surrounded by abnormal prominent enhancement in right Meckel’s cave and cavernous sinus (arrowhead).

He also had a high erythrocyte sedimentation rate, C-reactive protein level, and white blood cell count with a predominance of neutrophils. Moderate right-sided mixed-type hearing loss was evidenced on audiometry. Ear swab cultures were negative for microorganisms. Biopsy of his right external ear and mastoid cavity revealed only chronic inflammation.A CT scan of his temporal bone confirmed partially opacified right middle ear cavity and mastoid air cells, as a result of chronic otomastoiditis and nonpneumatization of right petrous (Figure 2A, see arrow) filled with fatty marrow, later confirmed by magnetic resonance imaging (MRI; Figure 2B). Subtle evidence of petrous apicitis of his right temporal bone was demonstrated by MRI (Figure 2C). Abnormal enhancement of the roof of right middle cranial fossa, the dura along the floor of the middle cranial fossa, thickened enhanced anteromedial part of right tentorial cerebelli and adjacent right cavernous sinus, as a result of superomedial extension of the infection, were demonstrated by MRI (Figure 3). Edematous right trigeminal ganglion within right Meckel’s cave (Figure 4), most likely to be responsible for his severe headache and facial pain, was also detected.

A 2-week course of intravenous ceftazidime (2g three times a day) and levofloxacin (750mg once a day) was commenced. Ofloxacin eardrops were continued for 2 weeks. Resolution of his headache was achieved after only 2 days of intravenous antibiotic administration. His primary position and right lateral gaze diplopia improved within 3 and 5 days, respectively. He was discharged and prescribed oral levofloxacin for 4 weeks. Complete recovery of his headache and right abducens nerve palsy occurred after 2 months and he is currently doing well.

## Discussion

GS is an uncommon but life-threatening complication of otitis media. The typical presentation of GS comprises a sixth cranial nerve palsy, otorrhea, headache, and pain along the distribution of the trigeminal nerve. Most cases of petrous apicitis do not present with the classic clinical triad, however [2,3]. The time interval between the onset of otitis media and the clinical presentation of abducens nerve palsy varies from 1 week to 2 to 3 months [4]. Most reported cases of GS in the last decade developed secondary to acute otitis media in children [1,2,4-7]. The present case is an unusual presentation of GS associated with previous history of radical mastoidectomy. Therefore, patients with a history of appropriate surgical management of chronic otitis media are potentially still at risk of developing GS.

In general, the petrous apex is composed of dense bone and bone marrow, as in our case. Thirty percent of petrous apex bones are well pneumatized and communicate with the middle ear cleft [8]. This anatomical variation allows susceptibility to similar pathological processes that can occur in the mastoid segment including obstruction, opacification, inflammation and infection. Our case highlights that lack of this anatomical communication does not exclude the potential for inflammation and infection at the petrous apex. The pathological mechanism of GS in our case was rare. The most plausible cause may have involved middle ear inflammation that extended to the dura via the defect in the tegmen tympani, resulting in local pachymeningitis that subsequently spread over the petrous apex. Inflammation that extends to the right cavernous sinus would involve the trigeminal and abducens nerves. The trigeminal nerve ganglia pass over the superior aspect of the petrous apex. The abducens nerve pierces the dura mater lateral to the dorsum sellae of the sphenoid. It bends sharply over the medial part of the petrous ridge of the temporal bone and passes forward through the inferior petrosal sinus in Dorello’s canal. This explains why cranial nerves V and VI can be easily affected by infection or inflammation of the petrous apex. The patient therefore developed sixth cranial nerve palsy and pain along the distribution of the trigeminal nerve ipsilateral to the site of the chronic otitis media, with subtle imaging evidence of petrous apicitis.

Other postulated mechanisms in the absence of petrous apicitis are phlebitis of the inferior petrosal sinus or venous spread of infection [1,9]. Microbiological studies of GS are difficult to perform, and cultures are frequently negative. Some studies have reported that the predominantly involved organisms include *Staphylococcus* species (spp.), *Pseudomonas aeruginosa*, Group A *Streptococcus* spp., and *Mycobacterium tuberculosis*[2].

A CT and MRI of the temporal bone are necessary and useful in evaluating structural abnormalities and differentiating the cause of GS. CT images typically show hypodensity at the petrous apex, whereas MRI shows hypointensity on T1-weighted images and hyperintensity on T2-weighted images. Both imaging modalities should reveal prominent enhancement in the area of infection or inflammation. MRI was useful to indicate the cause of GS in this patient by demonstrating pathologic changes that probably resulted from middle ear inflammation that extended via the tegmen tympani defect and caused pachymeningitis. The dura above the tegmen tympani is enhanced and extends to the right cavernous sinus. MRI is also beneficial in delineating the differential diagnosis of the many causes of GS, such as osteomyelitis, abscesses, cholesteatomas, neoplasms, and inflammatory granulomas [10,11]. Ibrahim *et al*. reported the use of diffusion-weighted MRI to demonstrate abscesses and cholesteatomas in regions of restricted diffusion [10].

Cases of GS as a complication of acute otitis media have usually been successfully treated with broad-spectrum antibiotics, even in cases of petrous abscess formation [10]. In the treatment of chronic ear disease, most authors support surgical intervention as primary management to ensure adequate petrous and mastoid drainage [5,12]. However, a case of successful conservative treatment of GS associated with chronic otitis media was reported with the use of antibiotic therapy [6]. Our case involved GS associated with chronic ear disease in a post-radical mastoidectomy patient successfully treated with conservative therapy. Although mastoid surgery was performed, inadequate surgery, loss to follow-up and infrequent postoperative mastoid cleaning may have been the causes of the chronically draining ear. A regular follow-up program should be encouraged to prevent this complication.

## Conclusions

We demonstrated a rare case of GS in spite of the clinical history of previous radical mastoidectomy and nonpneumatization of the petrous apex and the successful conservative treatment. A long-term follow-up program should always be encouraged in patients who have undergone radical mastoidectomy.

## Consent

Written informed consent was obtained from the patient for publication of this case report and any accompanying images. A copy of the written consent is available for the review by the Editor-in-Chief of this journal.

## Abbreviations

CT: Computed tomography; GS: Gradenigo’s syndrome; MRI: Magnetic resonance imaging; Spp.: Species.

## Competing interests

The authors declare that they have no competing interests. No author has any proprietary interest in any of the products or ideas mentioned in this article.

## Authors’ contributions

YP was a major contributor in writing the case report. YP and WK were equally responsible for data collection. WK and SH provided critical revision of the case report. All authors read and approved the final case report.

## References

[B1] Homer JJ Johnson IJ Jones NS Middle ear infection and sixth nerve palsy J Laryngol Otol 1996 110 872 874 10.1017/s0022215100135200 8949301

[B2] Lutter SA Kerschner JE Chusid MJ Gradenigo syndrome: a rare but serious complication of otitis media Pediatr Emerg Care 2005 21 384 386 10.1097/01.pec.0000166731.70847.d5 15942518

[B3] Tornabene S Vilke GM Gradenigo’s syndrome J Emerg Med 2010 38 449 451 10.1016/j.jemermed.2007.08.074 18296009

[B4] Scardapane A Del Torto M Nozzi M Elio C Breda L Chiarelli F Gradenigo’s syndrome with lateral venous sinus thrombosis: successful conservative treatment Eur J Pediatr 2010 169 437 440 10.1007/s00431-009-1047-4 19697060

[B5] Marianowski R Rocton S Ait-Amer JL Morisseau-Durand MP Manach Y Conservative management of Gradenigo syndrome in a child Int J Pediatr Otorhinolaryngol 2001 57 79 83 10.1016/s0165-5876(00)00442-0 11165646

[B6] Burston BJ Pretorius PM Ramsden JD Gradenigo’s syndrome: successful conservative treatment in adult and paediatric patients J Laryngol Otol 2005 119 325 329 10.1258/0022215054020313 15949093

[B7] Rossor TE Anderson YC Steventon NB Voss LM Conservative management of Gradenigo’s syndrome in a child BMJ Case Rep[published online April 20, 2011]. doi:10.1136/bcr.03.2011.3978 10.1136/bcr.03.2011.3978 PMC3082063 22696630

[B8] Gibier L Darrouzet V Franco-Vidal V Gradenigo syndrome without acute otitis media Pediatr Neurol 2009 41 215 219 10.1016/j.pediatrneurol.2009.03.008 19664541

[B9] Kong S-K Lee I-W Goh E-K Park S-E Acute otitis media-induced petrous apicitis presenting as the Gradenigo syndrome: successfully treated by ventilation tube insertion Am J Otolaryngol 2011 32 445 457 10.1016/j.amjoto.2010.07.018 20888067

[B10] Ibrahim M Shah G Parmar H Diffusion-weighted MRI identifies petrous apex abscess in Gradenigo syndrome J Neuroophthalmol 2010 30 34 36 10.1097/WNO.0b013e3181c5d0fd 20182204

[B11] Pedroso JL de Aquino CC Abrahão A de Oliveira RA Pinto LF Bezerra ML Gonçalves Silva AB de Macedo FD de Melo Mendes AV Barsottini OG Gradenigo's Syndrome: Beyond the Classical Triad of Diplopia, Facial Pain and Otorrhea Case Rep Neurol 2011 3 45 47 10.1159/000324179 PMC3072192 21490711

[B12] Minotti AM Kountakis SE Management of abducens palsy in patients with petrositis Ann Otol Rhinol Laryngol 1999 108 897 902 10.1177/000348949910800914 10527283

